# Polo-like Kinase 1 Inhibitors Demonstrate In Vitro and In Vivo Efficacy in Preclinical Models of Small Cell Lung Cancer

**DOI:** 10.3390/cancers17030446

**Published:** 2025-01-28

**Authors:** Guojing Zhang, Abbe Pannucci, Andrey A. Ivanov, Jeffrey Switchenko, Shi-Yong Sun, Gabriel L. Sica, Zhentao Liu, Yufei Huang, John C. Schmitz, Taofeek K. Owonikoko

**Affiliations:** 1 Division of Hematology and Oncology, University of Pittsburgh School of Medicine and UPMC Hillman Cancer Center, Pittsburgh, PA 15232, USA; abp78@pitt.edu (A.P.); zhl169@pitt.edu (Z.L.); yuh119@pitt.edu (Y.H.); 2 University of Maryland Greenebaum Comprehensive Cancer Center (UMGCCC), 22 South Green Street N9E17, Baltimore, MD 21201, USA; gzhang2@som.umaryland.edu (G.Z.); jschmitz@som.umaryland.edu (J.C.S.); 3 Department of Pharmacology and Chemical Biology, Emory University School of Medicine and Winship Cancer Institute, Emory University, Atlanta, GA 30322, USA; andrey.ivanov@emory.edu; 4 Biostatistics Shared Resource of Winship Cancer Institute, Atlanta, GA 30322, USA; jswitch@emory.edu; 5 Department of Hematology and Medical Oncology, Emory University School of Medicine and Winship Cancer Institute, Atlanta, GA 30322, USA; ssun@emory.edu; 6 Department of Pathology, University of Pittsburgh School of Medicine and UPMC Hillman Cancer Center, Pittsburgh, PA 15232, USA; sicagl@upmc.edu; 7 Cancer Virology Program, UPMC Hillman Cancer Center, Pittsburgh, PA 15232, USA

**Keywords:** small cell lung cancer, polo-like kinase, inhibitors, patient-derived xenografts, onvansertib

## Abstract

**Objective:** To investigate the preclinical efficacy and identify predictive biomarkers of polo-like kinase 1 (PLK1) inhibitors in small cell lung cancer (SCLC) models. **Methods:** We tested the cytotoxicity of selective PLK1 inhibitors (rigosertib, volasertib, and onvansertib) in a panel of SCLC cell lines. We confirmed the therapeutic efficacy of subcutaneous xenografts of representative cell lines and in four patient-derived xenograft models generated from patients with platinum-sensitive and platinum-resistant SCLC. We employed an integrated analysis of genomic and transcriptomic sequencing data to identify potential biomarkers of the activity and mechanisms of resistance in laboratory-derived resistance models. **Results:** Volasertib, rigosertib, and onvansertib showed strong in vitro cytotoxicity at nanomolar concentrations in human SCLC cell lines. Rigosertib, volasertib, and onvansertib showed equivalent efficacy to that of standard care agents (irinotecan and cisplatin) in vivo with significant growth inhibition superior to cisplatin in PDX models of platinum-sensitive and platinum-resistant SCLC. There was an association between *YAP1* expression and disruptive or inactivation *TP53* gene mutations, with greater efficacy of PLK1 inhibitors. Comparison of lab-derived onvansertib-resistant H526 cells to parental cells revealed differential gene expression with upregulation of *NAP1L3*, *CYP7B1*, *AKAP7*, and *FOXG1* and downregulation of *RPS4Y1*, *KDM5D*, *USP9Y*, and *EIF1AY* highlighting the potential mechanisms of resistance in the clinical setting. **Conclusions:** We established the efficacy of PLK1 inhibitors in vitro and in vivo using PDX models of platinum-sensitive and resistant relapsed SCLC. An ongoing phase II trial is currently testing the efficacy of onvansertib in patients with SCLC (NCT05450965).

## Simple Summary

We systematically examined the efficacy of three different polo-like kinase 1 (PLK1) inhibitors in preclinical models of small cell lung cancers (SCLCs). This study uncovered robust in vitro activity of PLK1 inhibitors against SCLC cell lines and confirmed the efficacy in patient-derived xenograft models of platinum-sensitive and resistant SCLC. Using mutation and transcriptomic profiling, we found a strong correlation of PLK1 inhibitor efficacy with inactivating *TP53* gene mutation and the expression of YAP1 transcription factor. The result of this preclinical study has been translated into the clinic in an ongoing phase II clinical trial of onvansertib in relapsed SCLC (NCT05450965) where YAP1 expression and inactivating *TP53* gene mutations will be interrogated as potential biomarkers to guide future clinical studies.

## 1. Introduction

Small cell lung cancer (SCLC) remains a significant health problem, with more than 180,000 newly diagnosed patients worldwide per year [1,2]. The outcome remains dismal, although the incorporation of immunotherapy into the standard frontline treatment has led to modest improvements [3,4,5]. Treatment options for this disease had not changed for three decades until the recent breakthrough in frontline chemoimmunotherapy [6,7]. Recurrence following initial frontline therapy is associated with resistance to salvage treatment and remains the greatest unmet need for patients [8,9,10,11,12].

Precision medicine approaches in the management of molecular subtypes of NSCLC with aberrant activation of kinase activity in *EGFR*, *ALK*, and *ROS1* among many others [13,14,15,16] have driven a significant decline in lung cancer mortality over the last two decades [5,17]. A personalized targeted treatment approach to guide SCLC management has yet to materialize despite the high rate of genomic perturbations, in part because the most common alterations occur in tumor suppressor genes (*TP53* and *RB1*) that are not yet targetable by pharmacological approaches [18,19,20,21]. Innovative strategies to exploit genetic alterations in SCLC for treatment guidance are urgently needed to bring the remarkable benefits of precision medicine to this population of patients.

The polo subfamily of serine/threonine (Ser/Thr) protein kinases, collectively referred to as polo-like kinases (PLKs), have been shown to be important regulators of cell cycle progression and cell proliferation [22,23,24]. Five members, PLK1, PLK2, PLK3, PLK4, and PLK5, exist in humans and exhibit differential functions and tissue distribution [23,25,26]. Dysregulation of this family of kinases is strongly associated with oncogenesis in human cells, including in lung cancer [22,23,27,28]. PLK1 is the most important and best-studied member of this family and is currently the target of pharmacological agents in clinical testing as anticancer therapy [29].

We identified PLK1 inhibitors as potential candidates for comprehensive preclinical evaluation based on low-throughput in vitro drug screening. We subsequently established the antitumor efficacy of a clinically relevant PLK1 inhibitor, onvansertib, using PDX models of SCLC. The SCLC YAP1 subtype may be preferentially sensitive to PLK1 inhibition. PLK1 inhibitor sensitivity was also correlated with disruptive p53 mutations, regardless of subtype. These identified potential predictive biomarkers informed the design of an ongoing clinical trial to test the clinical efficacy of onvansertib in SCLC (NCT05450965).

## 2. Materials and Methods

### 2.1. Reagents

Volasertib, rigosertib, and onvansertib were purchased from Selleckchem (Houston, TX, USA). The parent compound was dissolved in DMSO, aliquoted, and stored at −20 °C until ready for use in in vitro experiments or prepared fresh in PBS for xenograft experiments. Treatment-grade samples of cisplatin (Teva Parenteral Medicines, Inc., Irvine, CA, USA) and irinotecan were obtained from institutional outpatient pharmacies. The following antibodies were used for Western blotting: GAPDH (#sc-47724), TP53 (#sc-126) (Santa Cruz Biotechnology, Dallas, TX, USA); POU2F3 (#36135), ASCL1 (#10585), NEUROD1 (#7019), YAP1 (#14074), PLK1 (#4513), CMYC (#5605) (Cell Signaling Technology, Danvers, MA, USA).

### 2.2. Cell Lines and PDX Models

Human SCLC cell lines (H146, H187, H128, H69, H209, DMS153, H526, DMS114, and DMS53) were obtained from the American Type Culture Collection (ATCC, Manassas, VA, USA). Cell line purity was authenticated by STR profiling and mycoplasma infection was tested prior to in vitro and in vivo experiments. The growth conditions and maintenance of the cells have been described previously [30]. Briefly, cells were grown as a suspension or partially attached monolayer culture in RPMI 1640 medium (or Waymouth medium for DMS cells) along with FBS (5–10%) at 37 °C in a humidified atmosphere of 5% CO_2_ and 95% air. Previously generated PDX models of human SCLC (TKO-002, TKO-005, TKO-008, and TKO-010) were retrieved from our institutional tumor bank for in vivo experiments [31]. PDX models TKO-002, TKO-005, and TKO-010 were classified as the ASCL1 subtype, and TKO-008 was the NEUROD1 subtype.

### 2.3. In Vitro Cytotoxicity

Human SCLC cells were seeded in 96-well plates at approximately 0.5–3 × 10^4^ cells per well. After 24 h, exponentially growing cells were treated with continuous exposure to the vehicle or specific drugs of interest for 72 h. Cell proliferation was estimated using colorimetric assays (MTS (Promega, Inc., Madison, WI, USA) and WST1 (Takara Bio USA, San Jose, CA, USA)) according to the manufacturer’s protocol. The CellTiter-Glo 3D Cell Viability Assay (Promega) was used for densely clustered H526, H209, and H187 cell lines. Absorbance or luminescence was measured using a microplate reader (Infinite M1000 Pro, Tecan, Switzerland). The inhibitory concentration (IC_50_) of specific anticancer agents in the cell line was determined using GraphPad Prism software version 9 (GraphPad Software, Inc. La Jolla, CA, USA).

### 2.4. Western Blot Analysis

Preparation of whole-cell protein lysates and Western blot analysis were performed as described previously [30,32].

### 2.5. In Vivo Tumor Growth Inhibition

All animal experiments were conducted according to the requirements for humane treatment of animals under an animal protocol approved by the Institutional Animal Care and Use Committee. Tumor xenografts were raised in 6-week-old athymic (nu/nu) mice (Harlan Industries, Indianapolis, IN, USA). Animals were housed under pathogen-free conditions in microisolator cages and fed laboratory chow and water ad libitum. H526 cells (1−2 × 10^7^) suspended in serum-free medium were mixed with a Matrigel solution and injected subcutaneously into the flank region of nude mice. In vivo experiments using the PDX models were performed as previously described [31]. Subcutaneous tumor growth was measured until the tumor size reached approximately 100 mm^3^. Tumor-bearing mice were matched for body weight and tumor volume and randomly assigned to treatments at six–eight mice per group. Mice were administered volasertib (20 mg/kg; weekly intraperitoneally (IP)), cisplatin (3 mg/kg; weekly IP), irinotecan (25 mg/kg; weekly IP), rigosertib (250 mg/kg; daily IP), and onvansertib (60 mg/kg; orally daily for 10 days following a 4 day break). Tumor volumes (V = 1/2ab^2^, in which “a” and “b” represent length and width or tumor, respectively) and body weights were monitored twice a week. The subcutaneous tumors were harvested and weighed at the end of the experiment.

### 2.6. Whole-Transcriptome Profiling and Pathway Analysis

We accessed the gene expression data of human SCLC cell lines previously generated on the Illumina HT-12 platform and deposited in the NCBI Gene Expression Omnibus (GEO accession GSE55830) [30]. Raw probe intensities from untreated cell lines were normalized by the quantile normalization algorithm using GenomeStudio software version 2.0 from Illumina, and log-2 transformed expression was obtained for analyses. Supervised analysis was performed to cluster the cell lines by *MYC*, *PLK1*, and *TP53* gene expression. The IC_50_ concentrations of PLK1 inhibitors were compared between cell line clusters.

We also conducted differential gene expression analysis in SCLC cell lines based on data available through the Dependency Map (DepMap) Portal 21Q1 release [33]. Data from 48 SCLC cell lines were used, including 9 cell lines classified as YAP1 subtype (SCLC-Y) and 39 other subtype cell lines (not SCLC-Y). The log2(x + 1)-transformed RNA-seq TPM gene expression data were extracted from the CCLE_expression.csv file. The fold change was calculated between the average gene expression in the SCLC-Y and non-SCLC-Y groups. Cell drug sensitivity data were extracted from the Genomics of Drug Sensitivity in Cancer (GDSC) Portal [34]. *p* Values were calculated using *t*-tests. The false discovery rate-adjusted q-values were calculated using the Benjamini–Hochberg procedure.

### 2.7. Onvansertib-Resistant Cells

Onvansertib-resistant H526 cells were generated by growing H526 cells with increasing concentrations of onvansertib (10–75 nM) for over one year. Total RNA was extracted from parental and resistant cells at three separate times using the Qiagen RNeasy kit, and RNA-seq was performed using Novogene (Durham, NC, USA). The preprocessing steps involved the removal of adapter sequences and low-quality bases using Trimmomatic version 0.38. Subsequently, RSEM version 1.3.1 was employed to align the paired-end reads to the GRCh38 primary assembly, and the aligned mappings were further quantified into gene counts using the Gencode v43 primary assembly annotation. Differential gene expression analysis was performed using the DESeq2 package, which fits a negative binomial generalized linear model for each gene. The Wald test was used for significance testing. The results included estimated log fold changes, *p* values, and *p*-adjusted values. RNA-seq data were deposited in the NCBI Gene Expression Omnibus database (GEO accession GSE269636).

### 2.8. Statistical Analysis

Differences in mean IC_50_ concentrations of PLK1 inhibitors were compared for statistical significance by analysis of variance (ANOVA) or Kruskal–Wallis test, as applicable. The correlation between cell line sensitivity and degree of sensitivity to PLK inhibitors was measured using Pearson or Spearman correlation coefficients. The effects of treatment on tumor growth rate for a given treatment relative to the control group were determined as previously described using the formula %T/C = [(mean tumor volume of the treated group on day X divided by mean tumor volume of the control group on day X) × 100]. We assessed the differences in tumor volume and rate of tumor growth overall and by pairwise comparison between different treatment groups using a mixed-effect model. Overall and pairwise differences in the harvested tumor weights across the treatment groups were assessed for statistical significance using ANOVA. All analyses were performed using SAS 9.3 (SAS Institute, Inc., Cary, NC, USA) and GraphPad software with *p* < 0.05. Differential gene expression and cell drug sensitivity analyses were conducted in Python.

## 3. Results

### 3.1. Low-Throughput Screening for Targeted Agents in SCLC

**Figure 1:**
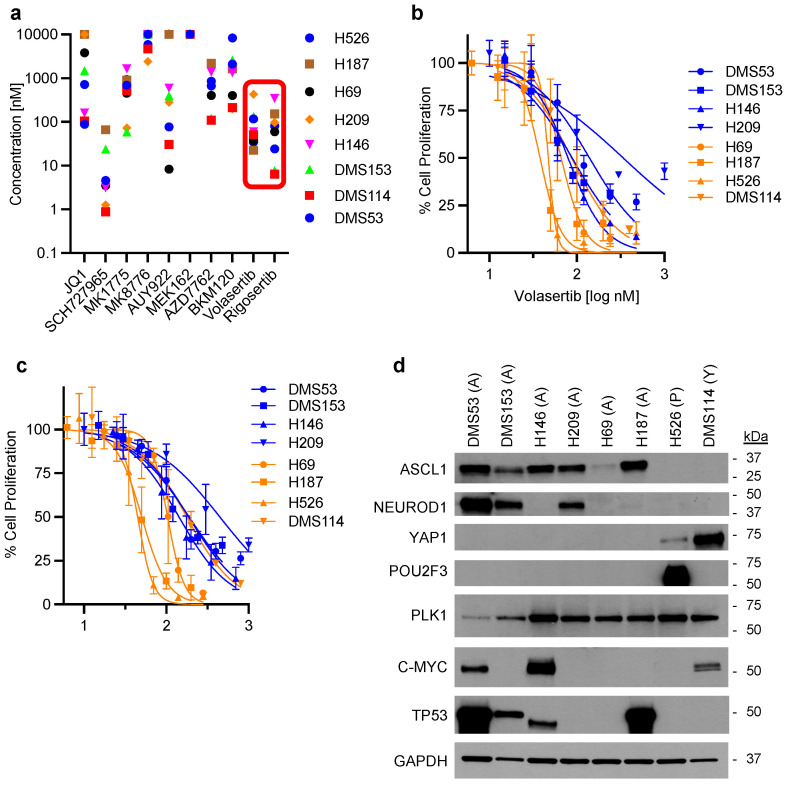
Assessment of in vitro antiproliferative activity of targeted agents in a panel of SCLC cell lines (**a**); effect of volasertib (**b**) and onvansertib (**c**) on proliferation of SCLC cell lines. Cells were treated for 72 h with indicated agents. Cell proliferation was determined using colorimetric or luminescent assays depending on the degree of clustering of SCLC cell lines in culture. Values represent the mean ± S.D. from a minimum of 3 independent experiments. Blue and orange curves define cell lines with non-disruptive and disruptive p53 mutations, respectively. Basal protein expression in SCLC cell lines (**d**). SCLC subtype based on expression are indicated after each cell line: ASCL1 (A), POU2F3 (P), YAP1 (Y).

To identify therapeutic leads for clinical studies in SCLC patients, we employed an agnostic low-throughput screening approach to evaluate in vitro cytotoxicity of targeted agents that have not been previously studied in SCLC human clinical trials. A priori, we established nanomolar in vitro cytotoxicity as the threshold required for any of the targeted agents to be considered worthy of detailed preclinical testing and eventual evaluation in patients. We established the in vitro cell proliferative activity of 10 different targeted agents in a panel of SCLC cell lines (H526, H187, H69, H209, H146, DMS153, DMS114, and DMS53), well characterized for genomic alterations, whole transcriptomic profiles, and transcription factor protein expression (Figure 1d). The initial panel of agents included compounds targeting HSP90 (AUY922), CHK1 (MK8776), CDK (SCH727965), MEK (MEK162), CHK1/2 (AZD7762), PIK3CA (BKM120), WEE1 (MK1775), PLK1 (rigosertib and volasertib), and bromodomain (BRD) (JQ-1) inhibitors. The activity of the agents targeting PLK1, CDK, and HSP90 met the a priori threshold of activity (Figure 1a). Some promising compounds were deprioritized because of concerns over their clinical safety profile (SCH727965) or lack of industry support for further clinical development (AUY922). PLK1 inhibitors were the most potent class of agents from this low-throughput screening, showing nanomolar activity of PLK1 inhibitors rigosertib and volasertib (BI6727) in the tested cell lines (Figure 1a,b). This observation, along with the biological role of PLK1 as an integration node for the cell cycle effects of RB1 and P53, informed the decision to focus further preclinical investigations on this class of agents. We subsequently tested a highly specific PLK1 inhibitor, onvansertib, against the same panel of cell lines and replicated the exquisite sensitivity of SCLC cell lines (Figure 1c).

### 3.2. In Vivo Activity of PLK1 Inhibitor Efficacy

**Figure 2:**
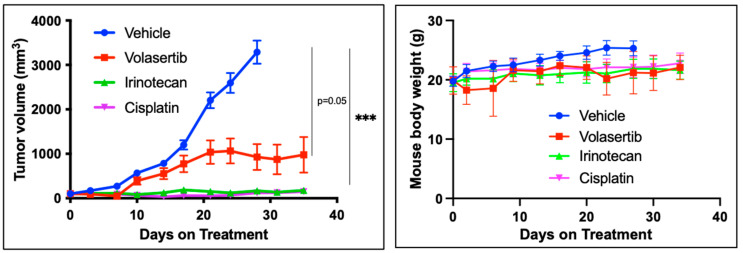
In vivo efficacy of PLK1 inhibitors in SCLC. Mice bearing H526 xenografts were i.p. administered volasertib (20 mg/kg), irinotecan (25 mg/kg), or cisplatin (3 mg/kg) weekly. Tumor volumes represent the mean ± SEM from groups of 6 mice. *** *p* ≤ 0.001.

**Figure 3:**
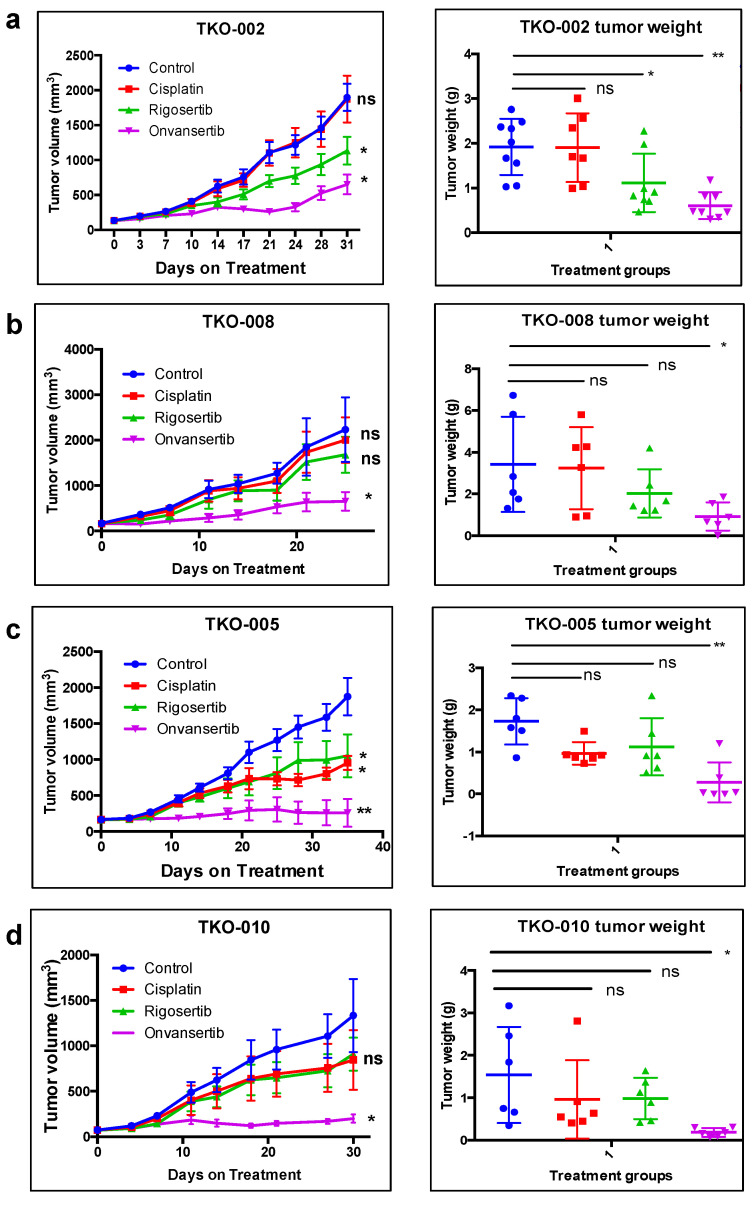
Antitumor efficacy of PLK1 inhibitors in SCLC PDXs. Mice bearing platinum-resistant PDXs TKO-002 and TKO-008 (**a**,**b**) and platinum-sensitive PDXs TKO-005 and TKO-010 (**c**,**d**) were administered cisplatin (3 mg/kg; i.p. weekly), rigosertib (250 mg/kg; i.p. daily), and onvansertib (60 mg/kg; oral × 10 days, 4 days off). Tumor volumes represent the mean ± S.D. from groups of 6 mice per group. *: significant and ns: not significant versus control group. * *p* ≤ 0.05, ** *p* ≤ 0.01.

We next wanted to establish whether the in vitro activity of PLK1 inhibitors translates into antitumor efficacy in vivo and how this activity compares with standard chemotherapy in patients with SCLC. We evaluated the antitumor efficacy of volasertib (20 mg/kg; weekly IP), cisplatin (3 mg/kg; weekly IP), and irinotecan (25 mg/kg; weekly IP) in a xenograft model of the H526 cell line, the most sensitive cell line for in vitro screening. Volasertib achieved significant tumor growth inhibition relative to the vehicle control in this model but was somewhat less effective than irinotecan and cisplatin (Figure 2). No major toxicity was observed when comparing changes in body weight between the treatment and control groups. SCLC PDX models are superior to traditional subcutaneous xenograft models for predicting clinical efficacy [35,36]. We previously demonstrated that the antitumor activity of rigosertib, another first-generation PLK1 inhibitor, was similar to that of cisplatin in a SCLC PDX model [31]. Therefore, we investigated the activity of PLK1 inhibitors in four different PDX models. In addition to the first-generation PLK1i, we evaluated onvansertib (NMS-P937, PCM-075, and NMS1286937), a highly selective, orally available PLK1 inhibitor with greater than 5000-fold selectivity for PLK1 over PLK2/PLK3 that is currently in clinical development [37]. Onvansertib has shown encouraging clinical efficacy as a single agent and part of a combination regimen for hematologic, colorectal, and prostate cancers [37,38,39]. Consistent with the results obtained with volasertib and rigosertib, onvansertib showed impressive in vitro activity at nanomolar concentrations against SCLC cell lines (Figure 1c). Onvansertib achieved significant tumor growth inhibition superior to cisplatin in PDX models of platinum-resistant (TKO-002 and TKO-008; Figure 3a,b) and platinum-sensitive (TKO-005 and TKO-010; Figure 3c,d) SCLC. There was no significant excessive toxicity, measured as body weight loss, in any of the tested PDX models.

### 3.3. Transcriptomic Analysis to Predict PLK1 Activity in SCLC

**Figure 4:**
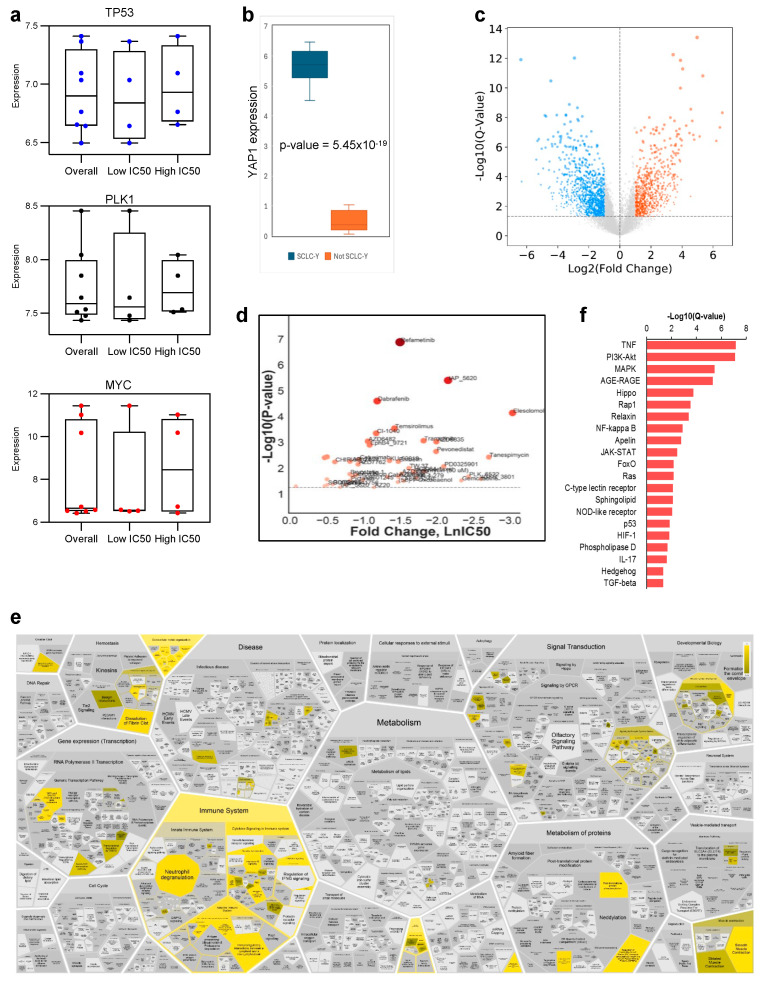
Correlative analysis between *TP53*, *PLK1*, and *MYC* expression (NCBI public database Gene Expression Omnibus GSE55830 [30]) and cell line sensitivity to PLK1 inhibition (**a**); YAP1 expression in SCLC-Y cell lines versus other subtypes (**b**); volcano plot of differentially expressed genes between SCLC-Y and not SCLC-Y cell lines (**c**); analysis of therapeutic vulnerability based on differential sensitivity of YAP1-positive cell lines showing PLK1 inhibitor as a potential candidate (**d**); Reactome analysis of active cellular function based on DEG between SCLC-Y and not SCLC-Y lines identified major differences in immune regulation and muscle contraction (**e**); KEGG analysis of differentially activated signaling pathways between the 2 groups (**f**).

Although PLK1 inhibitors showed potent in vitro activity at nanomolar concentrations against SCLC cell lines, there were differences in the IC_50_ concentrations between cell lines at the extremes of sensitivity (Figure 1b,c; Table 1). Using the median IC_50_ concentration for the entire panel of cell lines as a cut-off point, we observed a 10-fold difference in the mean IC_50_ concentration of volasertib (40 nM vs. 550 nM) in the most sensitive cell lines compared to that in the less sensitive cell lines (Table 1). Similar results were obtained using two other PLK1 inhibitors (onvansertib and rigosertib) in the two SCLC cell lines. To establish whether genotypic or phenotypic differences between the more sensitive and less sensitive SCLC cell lines could serve as predictive biomarkers of efficacy in patients, we investigated the transcriptomic profiles of the cell lines previously deposited in the NCBI public database Gene Expression Omnibus GSE55830 [30]. We correlated the normalized expression data of specific genes of interest with the degree of sensitivity to PLK1 inhibitors. Specifically, we compared the normalized expression of *TP53*, *PLK1*, and *MYC* between the more sensitive (Low IC_50_) and less sensitive (High IC_50_) lines. We found that the expression of these genes was not significantly different between the two groups in our cell line panel (Figure 4a). We probed the SclcCellMinerCDB database (68 SCLC lines) and correlated gene expression with volasertib sensitivity. PLK1 expression was not correlated with volasertib sensitivity (Supplemental Figure S1). Higher MYC expression tended to increase sensitivity to volasertib, whereas *TP53* expression was significantly elevated in cell lines most sensitive to volasertib.

The recently described transcriptional subtypes of SCLC are defined primarily by the selective enrichment of key transcription factors: achaete–scute homolog 1 (ASCL1), neurogenic differentiation factor 1 (NEUROD1), POU domain class 2 transcription factor 3 (POU2F3), and transcriptional coactivator YAP1 [40,41,42]. These subtypes also manifest key differences in their gene expression profiles. For instance, there is a higher expression of MYC in the non-neuroendocrine subtypes of SCLC, and MYC has been shown in murine models of SCLC to drive the transition from the neuroendocrine subtypes SCLC-A and SCLC-N to non-neuroendocrine SCLC-Y [43,44]. Therefore, we used publicly available datasets from the Genomics of Drug Sensitivity in Cancer (GDSC https://www.cancerrxgene.org/, accessed on 7 May 2021) and the Cancer Dependency Map (https://depmap.org/portal/, accessed on 6 May 2021) for an unbiased comparison of the therapeutic vulnerability of non-neuroendocrine SCLC-Y versus other SCLC subtypes (Figure 4b; Supplemental Table S1). We identified differentially expressed genes (DEG) that could indicate potential vulnerability in cell lines with high YAP1 expression (Figure 4c). Analysis of drug sensitivities between SCLC-Y and other subtypes of SCLC cell lines using the Cancer Therapeutics Response Portal database revealed specific drug candidates, including PLK1 inhibitors, that showed potent and highly selective activity in SCLC-Y cell lines (Figure 4d; Supplemental Table S2). This result further validated the initial results of our low-throughput screening strategy that identified PLK1 inhibitors as a promising class of agents. Intriguingly, gene set enrichment analysis (GSEA) using the Reactome pathway analysis tool [45] showed higher expression of genes involved in the immune system in YAP1-positive cell lines (Figure 4e). Similarly, KEGG database analysis of the DEGs revealed significant activity in various signaling pathways, including TNF, PI3K/AKT, MAPK, AGE-RAGE, and Hippo (Figure 4f).

### 3.4. TP53 Gene Mutation and PLK1 Inhibitor Efficacy

**Figure 5:**
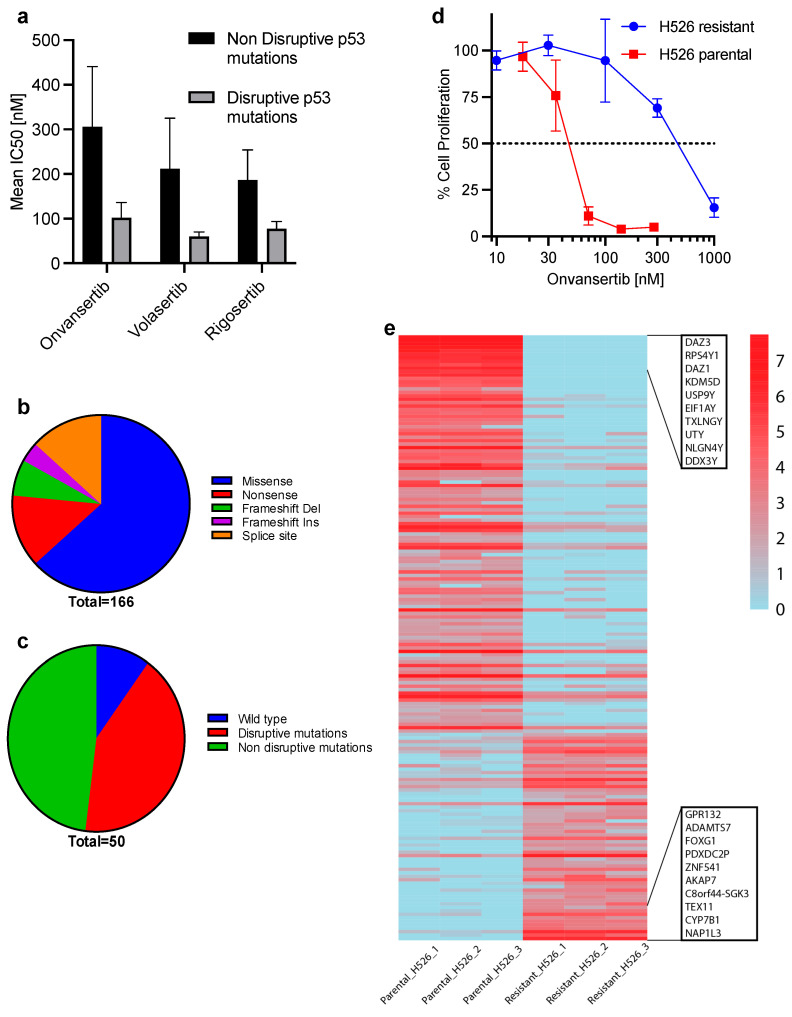
Effect of *TP53* mutational status on PLK1 inhibitor sensitivity. Comparison of mean IC_50_ to *TP53* gene mutation status (**a**). *TP53* gene status in 166 tumor samples in cbioportal.org (**b**) and 50 SCLC cell lines from publicly available CCLE data (**c**). Activity of PLK1 inhibitor onvansertib in parental and resistant H526 cells (IC_50_ concentration in the resistant vs. parent: 447 nM vs. 51 nM) (**d**). Gene expression profiles of matched parental and PLK1 inhibitor resistant H526 cells from 3 separate samples (**e**). Heatmap shows the top differential gene expression (*p*-adj < 0.5; logFC > 4 cut-off) with red indicating high and blue indicating low natural log-transformed expression.

Previous studies have shown that *TP53* mutations predict the sensitivity of cancer cell lines to PLK1 inhibition [46,47,48,49,50,51], and that cells with a normal diploid karyotype are insensitive to PLK1 depletion [48,49,52]. However, other reports have suggested no significant correlation between *TP53* mutations and sensitivity to PLK1 inhibition [53]. We posit that the discordant findings could be due to the failure to account for differences in the functional consequences of distinct types of *TP53* mutations. Therefore, we assessed the correlation between sensitivity to PLK1 inhibition and the predicted functional consequences of *TP53* gene mutations in our cell line panel. We correlated the IC_50_ of PLK1 inhibitors with genetic alterations that we previously established in our cell lines using a targeted DNA sequencing platform [30,54]. Overall, there was a high prevalence of *TP53* gene mutations in the cell lines (seven of eight cell lines), which is consistent with the published literature [18,19,20,21]. Intriguingly, the four cell lines (H526, H69, H187, and DMS114) with concomitant hemizygous deletion of *TP53* were more sensitive than the cell lines with wild-type or without concomitant hemizygous deletion (Table 1; Figure 5a). This result suggested that inactivation of *TP53* gene renders SCLC cell lines vulnerable to PLK1 inhibition, consistent with the published literature [51]. *TP53* gene mutation is a foundational genetic alteration present in nearly all SCLC cases. However, the functional consequences of these mutations differ based on the specific mutations and loci. We used publicly available Cancer Cell Line Encyclopedia databases to characterize the distinct types of *TP53* mutations in SCLC cell lines and tissue samples (https://www.cbioportal.org).

As expected, *TP53* mutations were highly prevalent but also varied by type and location (Figure 5b). Up to 60% of the alterations were non-disruptive, whereas 40% were disruptive inactivating mutations predicted to affect p53 function (Figure 5c). These results align with other published studies suggesting that total loss or inactivating mutation of *TP53* confers vulnerability to PLK1 inhibition, while a gain-of-function (GOF) mutation or WT status reduces sensitivity to PLK1 inhibitor [46,47,48,49,50,51]. Overall, our findings suggest that inactivating or disruptive *TP53* mutations confer vulnerability to PLK1 inhibitors, whereas GOF or non-disruptive mutations with retained DNA-binding function of p53 do not. We thus hypothesized that different *TP53* mutations in SCLC classified as disruptive (hemizygous deletion; truncation) or non-disruptive (in-frame; missense; GOF) mutations may be useful as predictive biomarkers for PLK1 inhibitor therapy.

### 3.5. Differentially Expressed Genes Associated with PLK1 Inhibitor Resistance

A known challenge for targeted therapies in the clinic is the inevitable development of resistance and loss of efficacy. Therefore, understanding the potential mechanisms driving acquired resistance could offer additional strategies to enhance the efficacy and delay the development of resistance or reverse acquired therapeutic resistance. We generated lab-derived PLK1i-resistant SCLC cells by continuous exposure of parental H526 cells to increasing concentrations of onvansertib (Figure 5d). Following confirmation of acquired resistance to onvansertib, the transcriptomic profiles of the parental and resistant lines were compared to identify differentially expressed genes. The onvansertib-resistant H526 cells showed reduced expression of *RPS4Y1*, *KDM5D*, *USP9Y*, and *EIF1AY* and increased expression of *NAP1L3*, *CYP7B1*, *AKAP7*, and *FOXG1* (Figure 5e; Supplemental Figure S2).

## 4. Discussion

SCLC is a major health challenge, with limited treatment options. Significant advances in targeted therapy options for non-small cell lung cancer over the past two decades have yet to materialize in SCLC, in part because of limited understanding of the disease biology and paucity of targetable genetic alterations. We investigated different classes of agents and uncovered potent in vitro cytotoxicity of PLK1 inhibitors against SCLC cell lines. We subsequently extended this finding to in vivo models, where we observed effective tumor growth inhibition by three different PLK1 inhibitors, namely, rigosertib, volasertib, and onvansertib. In particular, the activity of onvansertib against PDX models of platinum-sensitive and platinum-resistant SCLC highlights the clinical relevance of this study and supports the initiation of a clinical trial of onvansertib in patients with relapsed SCLC (NCT05450965).

We employed an agnostic low-throughput screening strategy focusing on targeted agents that can be easily tested in a clinical setting. While this approach limited the potential discovery breadth, the decision to narrowly focus the tested drug candidates on agents already in the clinic allowed for quick translation of the most promising findings into the clinical setting. Furthermore, while limiting the number of tested agents also introduced potential biases, the identification of PLK1 inhibitors as a promising class of agents and the potential role of YAP1 expression as a predictive biomarker in our work are consistent with independent work by others [40,55,56].

The identification of putative biomarkers to predict the benefit of PLK1 inhibitors in the clinical setting is of great value in the quest to bring precision medicine approaches to the management of SCLC. We did not observe any significant association at the transcriptomic level between the level of expression of PLK1 and the in vitro cytotoxicity of PLK1 inhibitors in our panel of SCLC cell lines. Intriguingly, however, loss-of-function mutation alone or in combination with hemizygous deletion in *TP53* gene was associated with greater efficacy. Our findings are consistent with previous studies suggesting that total loss or inactivating mutation of *TP53* confers vulnerability to PLK1 inhibition, whereas a gain-of-function mutation or preserved wild-type *TP53* activity reduces sensitivity to PLK1 inhibitor [46,47,48,49,50,51]. *TP53* is one of the most frequently mutated genes in human cancer. However, the frequency of *TP53* alterations is highly variable from one type of cancer to another, ranging from less than 5% in cervical carcinoma to 80–90% in SCLC [57,58,59]. P53 is a direct transcription activator for hundreds of genes but can also act as a transcriptional repressor [60]. Overall, non-synonymous single-nucleotide variants are the most common deleterious alterations in *TP53*, but other mechanisms, such as gene deletion, can also be present. Over 45,000 distinct *TP53* gene mutations have been reported and catalogued in various databases including 1540 single-nucleotide substitutions and 2000 frameshift mutants [60]. The location of a mutation event in the hot spot in exons 5–8, which encodes the DNA-binding domain of the *TP53* protein, drives the functional consequence. Disruptive mutations are non-conservative mutations within the key DNA-binding domain (L2 (codons 163–195)–L3 region (236–251)), or stop codons in any region, while the non–disruptive mutations are outside the L2–L3 region. Given the near-universal occurrence of *TP53* gene mutations in human SCLC and the obligatory requirement for *TP53* gene inactivation in the pathogenesis of murine SCLC [61], we proposed a concept to exploit the differential biology of inactivating versus gain-of-function *TP53* mutations to tailor therapy in SCLC. Finally, GSEA comparing resistance to parental cells showed strong modulation of immune-regulatory molecules in resistant cells. This is an interesting observation, and additional work beyond the current study is ongoing to determine whether a combination of PLK1 inhibitor and immune checkpoint antibodies could offer additional treatment strategies for this disease.

## 5. Conclusions

We identified PLK1 as a potential therapeutic candidate in preclinical models of SCLC. We established the promising activity of two different highly specific PLK1 inhibitors, volasertib and onvansertib, in vitro, in traditional xenograft models, as well as in PDX models of platinum-sensitive and resistant SCLC. We also uncovered the potential value of inactivating *TP53* gene mutations and high YAP1 expression as predictive biomarkers for patient selection in the clinic. This promising preclinical study is in clinical translation through a prospective phase II clinical trial of onvansertib in relapsed SCLC (NCT05450965). Furthermore, onvansertib is currently being clinically evaluated in combination with existing chemotherapies against multiple cancer types, including Kras^mut^-metastatic colorectal cancer, metastatic pancreatic cancer, and triple-negative breast cancer. Initial results from the randomized phase II mCRC trial (NCT05593328) report an objective response rate of 64%, which is significantly higher than with standard chemotherapy alone (33% ORR).

## Figures and Tables

**Table 1:** SCLC cell line sensitivity to PLK1 inhibitors measured by IC_50_ concentration.

Cell Line	Volasertib (nM)	Onvansertib (nM)	Rigosertib (nM)	*TP53* Gene Mutation Status	Hemizygous Deletion
DMS53	139.9 ± 21.8	188.9 ± 36.2	153.6 ± 23.7	c.722C > T	No
DMS153	90.0 ± 15.9	181.2 ± 36.8	114.8 ± 22.9	c.463A > C	No
H146	78.1 ± 14.5	145.3 ± 62.0	93.2 ± 16.9	Wild type	No
H209	550.7 ± 170.1	710.4 ± 260.0	385.7 ± 130.9	c.673-2A > T	No
H69 *	64.1 ± 19.9	105.1 ± 12.4	71.9 ± 19.0	c.511G > T	Yes
H187 *	40.4 ± 8.9	55.7 ± 19.3	61.8 ± 24.7	c.722C > G	Yes
H526 *	49.6 ± 14.3	51.4 ± 15.2	123.9 ± 32.6	c.97-1G > C	Yes
DMS114 *	87.1 ± 21.3	196.7 ± 58.6	52.3 ± 10.3	c.637C > T	Yes

*: indicates concurrent hemizygous deletion. In vitro cytotoxicity of PLK1 inhibitors against a panel of SCLC cell lines representative of different SCLC transcriptomic subtypes. Mean IC50 concentration for each compound and cell line was an average of 3–5 replicate experiments.

## Data Availability

The original data supporting the results presented in this manuscript generated in this study will be made available to interested researchers upon request, sent to the corresponding author and publicly at NCBI Gene Expression Omnibus (GEO accession GSE269636). Additional datasets analyzed during the current study are available in the NCBI Gene Expression Omnibus as series GEO accession GSE55830 [30], GEO accession GSE269636, Genomics of Drug Sensitivity in Cancer (GDSC; 7 May 2021) https://www.cancerrxgene.org/, and the Cancer Dependency Map (https://depmap.org/portal/, accessed on 6 May 2021).

## Supplementary Materials

The following supporting information can be downloaded at: https://www.mdpi.com/article/10.3390/cancers17030446/s1, Figure S1: Correlations between *PLK1*, *MYC*, and *TP53* gene expression and volasertib (ID#757149) sensitivity from SclcCellMinerCDB. Figure S2: Top 30 differentially expressed genes (DEGs) between resistant and parental cell lines based on *p*-value (**a**); top DEGs by GSEA Reactome Pathway analysis using a *p*-value < 0.01; FDR < 0.1 (**b**); top DEGs by GSEA Hallmark50 Pathway analysis using a *p*-value < 0.01; FDR < 0.1 as cut off (**c**). Table S1: Dominant level of YAP1 mRNA expression in cell lines included in the SCLC-Y versus other SCLC cell lines employed for comparative vulnerabilities to different anticancer agents using Cancer Therapeutics Response Portal database. Table S2: A list of the top differentially expressed genes and relevant therapeutic vulnerability in SCLC cell lines with high YAP1 expression in comparison to SCLC lines with low YAP1 expression using the publicly available Genomics of Drug Sensitivity in Cancer (GDSC) https://www.cancerrxgene.org/ and the Cancer Dependency Map (https://depmap.org/portal/).
