# Supplementary material for: Polo-like Kinase 1 Inhibitors Demonstrate In Vitro and In Vivo Efficacy in Preclinical Models of Small Cell Lung Cancer
Source: Cancers (Basel). 2025 Jan 28;17(3):446. doi: 10.3390/cancers17030446 (PMC11815996; doi:10.3390/cancers17030446)
Supplement: Supplementary file 1 [file cancers-17-00446-s001.zip › cancers-3406051-supplementary.pdf]

Supplemental Figure S1:

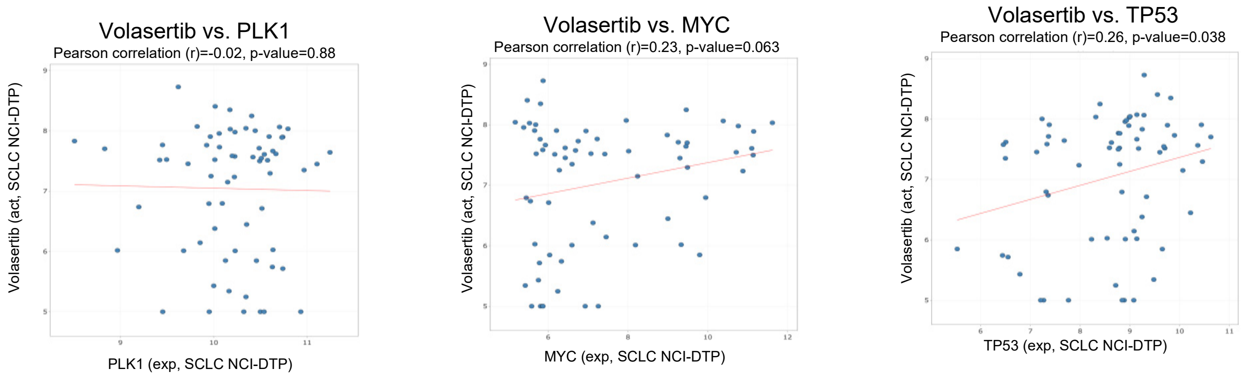

Correlations between *PLK1, MYC,* and *TP53* gene expression and volasertib (ID#757149) sensitivity from SclcCellMinerCDB.

Supplemental Figure S2:

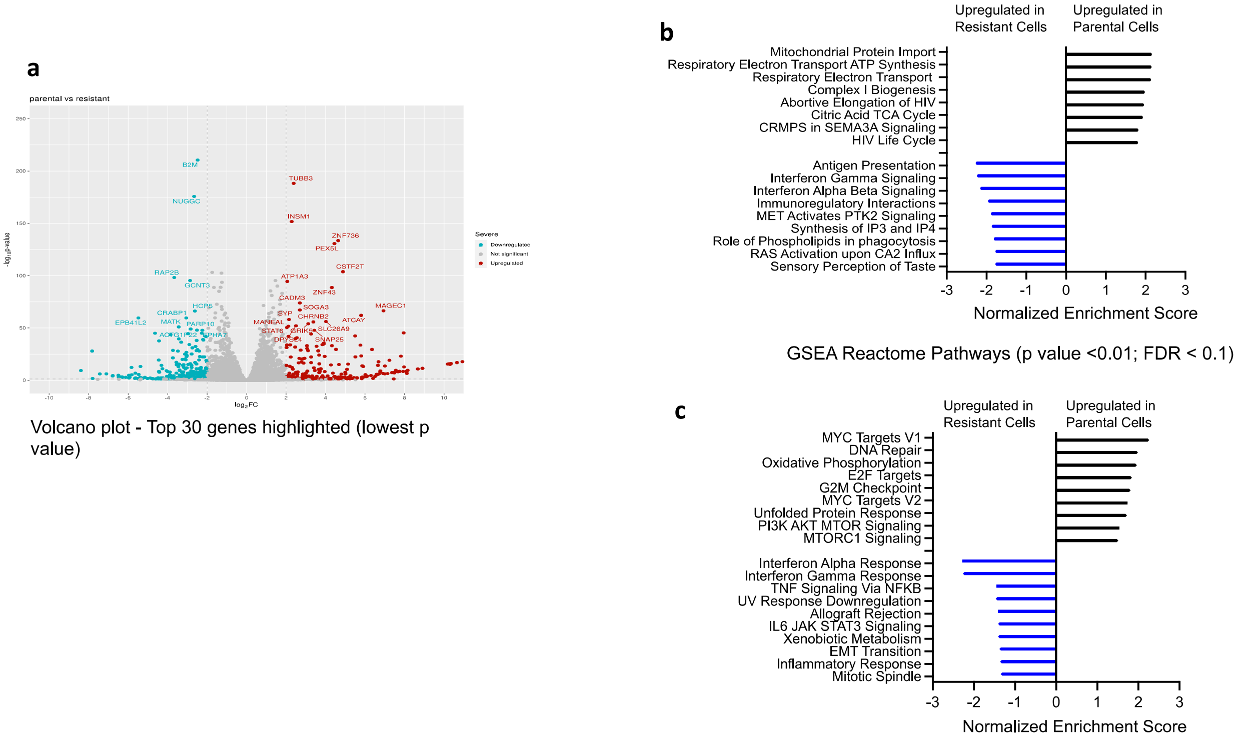

Top 30 differentially expressed genes (DEGs) between resistant and parental cell lines based on p-value (a); Top DEGs by GSEA Reactome Pathway analysis using a p-value <0.01; FDR < 0.1 (b); Top DEGs by GSEA Hallmark50 Pathway analysis using a p-value <0.01; FDR < 0.1 as cut off (c).

Supplemental Table S1:

	CELL	SUBTYPE	YAP1
ACH-000530	DMS114	YAP	5.27052894
ACH-000921	NCIH1339	YAP	5.85698569
ACH-000129	NCIH1341	YAP	5.11019618
ACH-000752	NCIH196	YAP	5.71121956
ACH-000912	NCIH2286	YAP	6.47021146
ACH-000292	NCIH841	YAP	6.15966955
ACH-000670	SBC5	YAP	5.45121111
ACH-000890	SW1271	YAP	7.72410452
ACH-000866	NCIH1048	YAP	4.51349075
ACH-000803	COLO668	NOTYAP	0.38956681
ACH-000695	CORL47	NOTYAP	0.36737107
ACH-000508	CORL88	NOTYAP	0.51601515
ACH-000743	CORL95	NOTYAP	0.41142625
ACH-000594	DMS153	NOTYAP	0.57531233
ACH-000844	DMS454	NOTYAP	2.54843662
ACH-000698	DMS53	NOTYAP	2.25096157
ACH-000703	DMS79	NOTYAP	0.37851162
ACH-000514	NCIH1092	NOTYAP	0.11103131
ACH-000780	NCIH1105	NOTYAP	0.20163386
ACH-000523	NCIH1184	NOTYAP	2.95419631
ACH-000830	NCIH1436	NOTYAP	0.26303441
ACH-000506	NCIH146	NOTYAP	0.05658353
ACH-000179	NCIH1618	NOTYAP	2.17632277
ACH-000559	NCIH1836	NOTYAP	0.29865832
ACH-000586	NCIH1876	NOTYAP	3.49569516
ACH-000870	NCIH1930	NOTYAP	0.89530262
ACH-000298	NCIH2029	NOTYAP	0.12432814
ACH-000394	NCIH2081	NOTYAP	0.80735492
ACH-000290	NCIH209	NOTYAP	0.20163386
ACH-000399	NCIH2196	NOTYAP	0.18903382
ACH-000871	NCIH510	NOTYAP	0.15055968
ACH-000177	NCIH660	NOTYAP	2.55090066
ACH-000358	NCIH69	NOTYAP	0.18903382
ACH-000297	NCIH889	NOTYAP	2.39231742
ACH-000790	SHP77	NOTYAP	0.23878686
ACH-000610	NCIH2227	NOTYAP	0.15055968
ACH-000257	CORL279	NOTYAP	0.57531233
ACH-000525	NCIH2171	NOTYAP	0.25096157
ACH-000659	SCLC21H	NOTYAP	0.68706069
ACH-000800	NCIH446	NOTYAP	0.15055968
ACH-000816	NCIH524	NOTYAP	0.13750352
ACH-000355	NCIH82	NOTYAP	0.20163386
ACH-000431	NCIH1694	NOTYAP	0.16349873
ACH-000382	CORL24	NOTYAP	0.62293035
ACH-000749	DMS273	NOTYAP	0.25096157
ACH-000187	CORL311	NOTYAP	0.422233
ACH-000639	NCIH211	NOTYAP	1.02856915
ACH-000767	NCIH526	NOTYAP	1.02147973

Dominant level of YAP1 mRNA expression in cell lines included in the SCLC-Y versus other SCLC cell lines employed for comparative vulnerabilities to different anticancer agents using Cancer Therapeutics Response Portal database.

Supplemental Table S2:

Gene Name	Protein name	Drugs
SIGMAR1	Sigma non-opioid intracellular receptor 1	Dextromethorphan;Fenfluramine;Pentazocine;Captodiame;Pentoxyverine
CAMLG	Calcium signal-modulating cyclophilin ligand	Cyclosporine
DHODH	Dihydroorotate dehydrogenase (quinone), mitochondrial	Leflunomide
CPT1A	Carnitine O-palmitoyltransferase 1, liver isoform	Perhexiline
UGCG	Ceramide glucosyltransferase	Miglustat;Eliglustat
SHBG	Sex hormone-binding globulin	Tamoxifen
SERPIND1	Heparin cofactor 2	Ardeparin;Sulodexide
CCND1	G1/S-specific cyclin-D1	Arsenic trioxide;Encorafenib
CDK6	Cyclin-dependent kinase 6	Palbociclib;Ribociclib;Abemaciclib
FADS2	Fatty acid desaturase 2	alpha-Linolenic acid
ITGAV	Integrin alpha-V	Antithymocyte immunoglobulin (rabbit);Levothyroxine
SCN1B	Sodium channel subunit beta-1	Zonisamide
GANAB	Neutral alpha-glucosidase AB	Miglitol
SCN1B	Sodium channel subunit beta-1	Zonisamide
SIRT2	NAD-dependent protein deacetylase sirtuin-2	Cambinol
GRIK5	Glutamate receptor ionotropic, kainate 5	Topiramate

A list of the top differentially expressed genes and relevant therapeutic vulnerability in SCLC cell lines with high YAP1 expression in comparison to SCLC lines with low YAP1 expression using the publicly available Genomics of Drug Sensitivity in Cancer (GDSC) https://www.cancerrxgene.org/ and the Cancer Dependency Map (https://depmap.org/portal/).
